# Phyto-Friendly Soil Bacteria and Fungi Provide Beneficial Outcomes in the Host Plant by Differently Modulating Its Responses through (In)Direct Mechanisms

**DOI:** 10.3390/plants11202672

**Published:** 2022-10-11

**Authors:** Monica De Palma, Riccardo Scotti, Nunzio D’Agostino, Massimo Zaccardelli, Marina Tucci

**Affiliations:** 1 Institute of Biosciences and BioResources, Research Division Portici, National Research Council, 80055 Portici, Italy; 2 CREA Research Centre for Vegetable and Ornamental Crops, Via Cavalleggeri 25, 84098 Pontecagnano Faiano (SA), Italy; 3 Department of Agricultural Sciences, University of Naples Federico II, 80055 Portici, Italy

**Keywords:** rhizobiome, gene expression, beneficial soil microbes, plant growth, induced systemic response

## Abstract

Sustainable agricultural systems based on the application of phyto-friendly bacteria and fungi are increasingly needed to preserve soil fertility and microbial biodiversity, as well as to reduce the use of chemical fertilizers and pesticides. Although there is considerable attention on the potential applications of microbial consortia as biofertilizers and biocontrol agents for crop management, knowledge on the molecular responses modulated in host plants because of these beneficial associations is still incomplete. This review provides an up-to-date overview of the different mechanisms of action triggered by plant-growth-promoting microorganisms (PGPMs) to promote host-plant growth and improve its defense system. In addition, we combined available gene-expression profiling data from tomato roots sampled in the early stages of interaction with *Pseudomonas* or *Trichoderma* strains to develop an integrated model that describes the common processes activated by both PGPMs and highlights the host’s different responses to the two microorganisms. All the information gathered will help define new strategies for the selection of crop varieties with a better ability to benefit from the elicitation of microbial inoculants.

## 1. Introduction 

Sustainable intensification that limits the ecological footprint is one of the main challenges humans will face in agriculture. The growing demand to reduce the use of chemicals is currently leading to a greater focus on more environmentally friendly crop management [1].

Beyond the well-known mycorrhizal fungi and Rhizobium bacteria, other non-symbiotic rhizobacteria (PGPR) and fungi (PGPF) are recognized as plant-growth-promoting microorganisms (PGPMs). A large amount of commercial PGPM-based products are currently available and used in organic and integrated agriculture for their ability to increase plant development and growth and improve plant health by strengthening crop resistance to (a)biotic stresses [2,3,4]. Commercial microbial consortia, generally consisting of a mix of rhizosphere bacteria (mainly *Pseudomonas* spp. and *Bacillus* spp.), useful fungi (*Trichoderma* spp.), nitrogen-fixing bacteria (principally *Azotobacter* spp. and *Azospirillum* spp.), or arbuscular mycorrhizal fungi, are receiving considerable attention for their additive/synergistic biostimulant effects on inoculated plants [5]. Microbial consortia can be designed as agricultural probiotics, as they stimulate the recovery of functional, beneficial microbial groups and supply the natural microbiome reduced by crop domestication [6]. Furthermore, they are used in plant microbiome engineering to modify the structure of pre-existing microbial communities [7].

Despite the above-mentioned beneficial effects, some limitations need to be overcome for large-scale exploitation of PGPMs in agriculture [3,8]. Indeed, PGPM use is still quite limited because of (i) the low availability of high-throughput methods for their screening, isolation, and characterization; (ii) constraints in the large-scale production technologies at industrial level; (iii) the lability of the formulations on the market and the few efforts in producing innovations related to the improvement of the shelf-life of PGPM formulations; (iv) the dearth of innovative and effective strategies and methods for their delivery (i.e., the development of adequate and reliable carriers based on the most advanced nanobiotechnologies) [9,10]; (v) the lack or ineffectiveness of regulations and guidelines that restrict their commercialization [11]; and (vi) the small number of well-trained operators responsible for quality control and biosecurity of the formulations. Nevertheless, the number of agrochemical companies and start-ups developing and marketing microbial products has significantly increased in recent years [12].

Although the use of PGPMs is having great success in innovative agricultural practices and considerable progress has been made in characterizing the complexity of plant–PGPM interactions, there are still several open questions regarding how microbial assembly is established, developed, and maintained throughout the plant’s life cycle. High-throughput technologies and ‘omics’ sciences have accelerated the understanding of plant relationships with microbial communities and especially of (i) how plants and microbes intimately interact, and (ii) how microbes affect plant development, fitness, health, and adaptation to environmental perturbation, (a)biotic stress susceptibility/tolerance, nutrient transfer, cycling, etc. [13,14]. In recent years, huge strides have been made in the field of metagenomics, hence allowing us to identify and characterize mutualistic and antagonistic plant microbial communities starting from the DNA directly extracted from an environmental sample [15].

RNA-based studies are the basic requirement to study the temporal evolution of the complex interaction process between the host plant and the microbes. At present, most reports only focus on host transcriptomics, whose goal is to investigate the transcriptome dynamics and derive gene expression patterns to identify the key processes activated in the plant following the beneficial interaction with soil microbes and to describe the plant adaptation response [16,17,18,19,20].

Dual RNA-sequencing or multi-species transcriptomics, that is the parallel analysis of transcriptome dynamics and gene expression profiles of microbes and their hosts, is much less usual despite being essential to reveal the molecular mechanisms that mediate host/microbe interplay [21,22].

A further level of analysis concerns the identification and quantification of the chemical compounds and the characterization of the metabolic alterations that occur during host–microbe communication [23]. Indeed, metabolomics is increasingly used to unravel the chemical complexity underlying the interactions between plant hosts and their associated microbial communities [24]. Knowing what metabolic changes are taking place is essential to identify which plant metabolites are key players in favoring the interaction with the rhizosphere microbiome, which microbe chemical classes are necessary to establish an effective cooperation relationship and ultimately to reconstruct metabolic models for specific communities of interactors. The next step is to integrate data and knowledge across the omics cascade to investigate plant–microbe relationships at multiple biological scales, moving from the gene to ecosystem levels. The complementation of omics data (i.e., holomics) allows us (i) to get a more precise overview and a systems-level understanding of the plant holobiont (namely, the plant and its associated microbes), (ii) to unlock the molecular reprogramming at play in both host and microbe community during the interaction, and (iii) to resolve the structure and functioning of agricultural ecosystems [25].

The ultimate goal is to move from basic to applied research, i.e., to manipulate these interactions to make them even more efficient. This means, on the one hand, improving plant health and increasing productivity and, on the other, developing sustainable disease management strategies, remodeling the microbiome in soil systems, promoting soil health and ecosystem homeostasis, and fostering the sustainability of ecosystems.

The main objective of this review is to provide an overview of the most up-to-date knowledge on how host plants differently modulate the cascade of events and processes following stimulation with bacteria or fungi. To achieve our goal, we review the recent literature and studies describing the (in)direct mechanisms of action promoted by PGPMs to stimulate plant growth and improve their tolerance to (a)biotic stresses. In addition, we present a case study based on recently published gene expression profiling datasets (i.e., tomato-*Pseudomonas fluorescens* and tomato-*Trichoderma harzianum*) to develop a model that describes the common and distinctive molecular responses of the host plant in the early stages (24, 48, and 72 h) of the PGPM interaction [17,18]. All the information gathered will help in defining new strategies for the selection of crop varieties with a better ability to benefit from the elicitation of microbial inoculants. 

## 2. Overview of the Mechanisms Modulated by PGPMs to Improve Plant Growth

Most of the PGPMs that populate the rhizosphere [26] are associated with the root system and exert a phytostimulating effect, attributable to both direct and indirect mechanisms. Direct mechanisms include nutrient availability in soil (e.g., solubilization and mobilization of nutrients) and the production of phytohormones [27,28]. The exploitation of lytic enzymes, antibiotics, siderophores, and volatile and non-volatile metabolites, as well as the induction of systemic resistance (ISR) for the suppression of harmful phytopathogens, have been reported as the main indirect mechanisms through which PGPMs promote plant growth and development [29,30]. 

Hydrogen cyanide (HCN) is a volatile secondary metabolite synthetized by several PGPMs [31,32] that can control the level of deleterious microbes in the rhizosphere. For example, the suppression of tomato root knot disease caused by *Meloidogyne javanica* is mediated by HCN-producing *P. fluorescens* [33]. Recently, a large body of evidence has demonstrated that PGPM stimulation of plant growth results also from complex chemical signaling mediated by volatile organic compounds (VOCs) [34,35]. VOC production is widespread and can be very different between PGPR and PGPF [36]. VOCs produced by both *P. fluorescens* SS101 and *B. subtilis* SYST2 increased plant biomass [34,37]. Plant exposure to VOCs emitted by different *Trichoderma* spp. was found to significantly improve the chlorophyll content, size, and biomass of plants [38]. 

Table 1 lists some examples of growth benefits conferred to plants by different PGPMs, as well as the various mechanisms involved.

## 3. Direct Mechanism of Plant Stimulation by PGPMs

### 3.1. Role of PGPMs in the Availability of Soil Nutrients and in the Uptake of Nutrients 

Plant nutrition is essential for optimal agricultural production, thus an adequate supply of nitrogen (N), phosphorus (P), and sulfur (S) forms and ionic species is necessary and usually added to the soil as synthetic fertilizers [58]. However, the increase in the use of chemical fertilizers does not proportionally guarantee crop yields and has a long-term impact on the environment in terms of water and soil pollution, depletion of soil fertility, and carbon footprint [59]. Phyto-friendly soil microbes are a very promising alternative to conventional fertilizers [60,61]. 

More than a half of the N released into the soil by the conventional N-based fertilizers is lost through the processes of denitrification, runoff, leaching, and erosion. Despite the abundance of nitrogen in the Earth’s atmosphere, the gaseous form of nitrogen is not readily accessible to plants until its conversion into ammonia [62]. In this context, PGPMs can support nitrogen assimilation mainly through mineralization and nitrogen fixation [63], hence enhancing N use efficiency [49]. For example, free-living, associative, and endophytic nitrogen-fixing bacteria have been found to increase plant growth, vigor, and yield in various non-leguminous crops such as rice (Table 1), wheat and sugarcane [39,64]. Recently, fertilizer formulations that included free-living N_2_-fixing bacteria along with organic and inorganic N forms proved to be a promising strategy for reducing N leaching and improving growth in sugarcane and macadamia plants [65,66]. The presence of fungal inoculants, mostly belonging to the genus *Trichoderma* (Table 1), optimizes nitrogen-use efficiency (NUE) in lettuce (*Lactuca sativa* L.) and rocket (*Eruca sativa* Mill.), favoring the uptake of the native N of the soil and improving N uptake by roots in conditions of scarce availability [48,49]. Inorganic N in the form of nitrate (NO_3_^−^) and ammonium (NH_4_^+^) can be uptaken by plants directly from the soil because of the activation of nitrate (NRT)/ammonium (AMT) transporters, which have been thought to be the key components for improving NUE [67,68,69]. Recently, Calvo et al. [70] found that root stimulation with mixtures of PGPR belonging to the genus *Bacillus* increased nutrient uptake in *Arabidopsis thaliana,* affecting the transcriptional levels of both NRT and AMT transporters. Otherwise, following the stimulation of tobacco roots with *T. asperellum*, the increased expression of several high-affinity *NRT* genes and the down-regulation of the *AMT1* gene suggested a preferential induction of the NRT transporter system for nitrate acquisition [71]. 

Despite being present in large amounts in the soil, P is a major plant-growth-limiting nutrient because inorganic phosphate (orthophosphate; Pi), the chemical form that can be assimilated by the plant, is available at low concentrations [72]. The mechanisms involved in the solubilization/mineralization of P from inorganic and organic forms in soil are well-documented in PGPMs [73]. In this regard, several PGPMs have been shown to enhance P assimilation and, as a consequence, growth and the yield when applied to crops (Table 1). *Paenibacillus* and *Trichoderma* are promising candidates for crop inoculation because of their ability to solubilize Pi mainly through the acidification of the soil environment by the production of organic acids [74,75]. Plant acquisition of Pi and its homeostasis is mediated by phosphate transporters (PTs) [72] and PGPMs could favor these processes through the transcriptional modulation of members in the PT gene family [76]. Recently, attention to the regulatory effects of PGPR on PT genes has been reported for the phosphate-solubilizing rhizobacterium *Pseudomonas* sp. P34-L. This strain significantly increased P accumulation in wheat and this ability correlates with reduced transcript levels of the *TaPT4* gene as an indicator of phosphorus deficiency [77]. 

Finally, a greater availability of many nutrients such as calcium, magnesium, sulfur, iron and zinc is necessary for the development of physiological and metabolic processes in plants [78]. In a low-nutrient-intake scenario, the zinc solubilization activity of PGPR (Table 1) has been reported as a new sustainable approach to overcome zinc deficiency and increase plant growth [43]. Interestingly, *T. harzianum* T-203 (Table 1) increased the concentrations of several macro- and micro-nutrients (e.g., P, Fe, and Zn) in the roots of cucumber, through the mineralization of organic matter in the soil [51]. 

### 3.2. Role of PGPMs in Hormonal Balance 

Colonization of the rhizosphere is known to be associated with profound changes in hormone homeostasis. Phytohormones act as messengers to coordinate cellular activities and to regulate plant growth, modification of root and shoot architecture, and synthesis of secondary metabolites [79,80,81]. Auxins are an important class of phytohormones that influence the size of shoot and root meristems [82]. Phyto-friendly soil microbes can directly affect plant auxin metabolism (Table 1) by synthesizing auxins [83] or indirectly by influencing the level of endogenous plant auxin [46]. 

Several studies involving different strains of *Aeromonas punctata*, *Azospirillum brasilense*, and *Burkholderia cepacian* suggest that auxin synthesis may be the main cause of the stimulating effect of some PGPR strains on host plants [80,84], while some PGPF (Table 1) can promote root growth also by influencing the level of endogenous plant auxin [56]. 

Since PGPMs can have both beneficial and harmful effects on the plant depending on their quantity and environmental conditions, the plant can perceive them as weak biotrophic pathogens. The synthesis of auxins by PGPR may be part of a strategy to suppress the resistance of the host allowing for a more effective colonization. Therefore, the ability to synthesize auxins and their quantity are important characteristics of a PGPR strain, as they can determine the strain’s impact on the plant to a large extent. PGPR can also influence auxin transport by altering the activity of auxin influx and efflux carriers. For example, *Bacillus* sp. LZR216 negatively regulates the synthesis of auxin transporters AUX1 and PIN1, -2, and -3 [45], while treatment with *Burkholderia phytofirmans* PsJN results in greater expression of PIN2 and PIN3 [46].

Meents et al. [53] reported that the exposure of *A. thaliana* to *Piriformospora indica* (Table 1) significantly increases auxin levels and induces the expression of auxin-responsive genes in lateral root primordia and root elongation zone within 1 day. Elevated auxin levels were also recorded in the *Mortierella hyalina*/*Arabidopsis* root interaction, but no downstream effects were observed on the auxin-responsive genes [53]. Some strains of *T. virens* and *T. atroviride* are known to produce indole-3-acetic acid (IAA) and auxin compounds that affect plant growth and root development [56,85], resulting in greater nutrient absorption efficiency. On the other hand, some secondary metabolites can act as auxin-like molecules. Vinale et al. [54] reported that 6-n-pentyl-6H-pyran-2-one (6PP) from *Trichoderma* can be considered an auxin-like compound or can act as an auxin inducer.

Another important phytohormone affected by PGPMs is ethylene. It plays a key role in plant growth and developmental processes such as ripening, senescence, and abscission, and in regulating the defense response of plants to various (a)biotic stresses [86]. Phyto-friendly soil microorganisms can influence plant ethylene homeostasis (Table 1) by affecting the expression of the genes that encode for enzymes responsible for the synthesis of ethylene such as ACC-synthase (ACS) and ACC-oxidase (ACO) [46,87], or by expressing ACC-deaminase, thereby reducing the amount of ethylene in the plant by degrading its precursor, 1-aminocyclopropane-1-carboxylic acid (ACC) [88,89]. The resulting changes in ethylene levels can affect root system development. Mayak et al. [90] reported an increase in the number of roots in mung bean plants treated with two strains of *Pseudomonas putida* that produce IAA. 

The ability to produce ACC-deaminase is also present in PGPF, as in the case of *T. asperellum*, which exhibits a reduced ability to promote root elongation of canola seedlings when a specific ACC gene is knocked out [55]. PGPMs with ACC-deaminase activity are known to mitigate the damaging effects of abiotic stresses on plant growth and development, such as high salinity, soil pollution by heavy metals and organic pollutants, floods, drought, and mineral deficiency [16,91]. Treatment with bacterial consortia (*Aneurinibacillus aneurinilyticus* and *Paenibacillus* spp.) significantly reduced (∼60%) stress-stimulated ethylene levels (Table 1) and its associated growth inhibition in *Allium sativum* L. [47]. In Brotman et al. [16], ACC-deaminase-silenced *Trichoderma* mutants were less effective in providing salt-stress tolerance, suggesting that *Trichoderma*, similarly to bacteria that produce ACC deaminase, can promote plant growth under abiotic stress, lowering ameliorative increases in ethylene levels and promoting high antioxidant capacity.

## 4. ISR as Indirect Plant Defense Mechanisms Mediated by PGPMs to Promote Plant Growth

The complex microbial community of the plant rhizosphere is critical to support the defense response of crops against a wide range of pathogens. In addition to direct defense mechanisms (e.g., antagonism, competition) [92,93], ISR is an important indirect mechanism by which PGPMs can help plants by improving their physical, chemical, and molecular defense responses [94]. All these activities have a positive effect on plant physiology and, indirectly, on plant-growth promotion. ISR is activated by the PGPMs of the rhizosphere via a mobile root-to-shoot signal involving different elicitors such as lipopolysaccharides, VOCs, or the regulation of hormone balance [95,96]. VOCs produced by PGPMs, in addition to plant-growth-promoting activity, can control pathogens in the rhizosphere through antifungal and antibacterial activities and have long-range control capabilities because of their volatile nature [97,98]. 

Unlike systemic acquired resistance (SAR), ISR has been found to act through ET- and JA-dependent pathways to confer systemic protection against pathogens and pests [95] and results in the strengthening of structural barriers, increasing antioxidant levels, and affecting the defense-related gene expression [95,99]. 

Table 2 lists some examples of growth benefits conferred on plant defense by different PGPMs and the various mechanisms involved. 

### 4.1. Strengthening of Cell Wall and of Plant Defensive Potential

The cell wall plays a key role in determining cell shape and size, and in controlling cell differentiation and growth, and represents the main dynamic barrier against pathogen attack [123]. In the early stage of plant/phyto-friendly soil bacteria interaction, changes in lignin content and alterations of chemical composition of the cell wall were detected in roots [124,125]. Similarly, pretreatment of roots with two different *Trichoderma* species resulted in a reorganization of cell wall components, such as pectin rearrangement, accumulation of lignin, and arabinogalactan proteins [126]. The strengthening and remodeling of the cell wall are considered some of the underlying mechanisms of ISR mediated by PGPMs to improve plant resistance to pathogens. For example, plants inoculated with *B. pumilus* or different *Trichoderma* strains showed increased lignin and callose deposition in the cell wall (Table 2) and these modifications were able to prevent the growth and proliferation of the invading pathogens more rapidly in plants inoculated with PGPMs than uninoculated plants [100,111]. 

It is well-documented that the phyto-friendly soil microbes stimulate defense responses of host plant through the activation of defense-related enzymes (e.g., phenylalanine ammonia-lyase (PAL), chitinase, β-1,3 glucanase, etc.) as well as of an enzymatic and non-enzymatic defense mechanism that efficiently scavenge reactive oxygen species (ROS) [127]. *P. aureofaciens* 63–28 increased the activity of PAL, peroxidase (POX) and polyphenol oxidase (PPO) enzymes (Table 2) in cucumber root challenged by *P. aphanidermatum* [101]. In chilli pepper plants, pretreatment with different PGPF (Table 2) induced resistance against the pathogen *C. truncatum* by enhancing the activity of defense (e.g., PAL, PPO), as well as of antioxidant enzymes (e.g., catalase, ascorbate peroxidase) [114]. Interestingly, the two pathways of the ascorbate–glutathione cycle (Asa-GSH) and the oxidative pentose phosphate (OPP) involved in the regulation of efficient redox homeostasis have recently been shown to participate in the enhanced defense response induced by *T. harzianum* (Table 2) in cucumber roots challenged by *F. oxysporum* [116].

Secondary metabolites with antioxidant and antimicrobial properties such as phytoalexins and flavonoids are involved in the ISR defense mechanisms. Induction of ISR by some *Pseudomonas* strains (Table 2) protected plants from different pathogens and pests through strong activation of phytoalexins/glucosinolates biosynthetic pathways [104,105]. A genome-wide characterization of the ISR induced by *T. hamatum* T382 in *A. thaliana* resulted in the up-regulation of genes in the phenylpropanoid pathway (Table 2) leading to the production of flavonoids (mainly anthocyanins) which may play a key role in *Trichoderma*-mediated antioxidant defense against the necrotrophic pathogen *B. cinerea* [117].

### 4.2. Hormones and PGPM-Induced Systemic Resistance

PGPMs stimulate the immune response of host plants against soil-borne diseases and pests [97]. However, from the plant’s point of view, the PGPM is an alien organism and the plant recognizes it as a pathogen, thus activating an immune response, which the PGPM must then overcome to establish a successful interaction. Since PGPMs live in close association with the plant, the colonization process is similar to the infection by biotrophic pathogens and, therefore, the initial defense response against these two classes of microorganisms is comparable [128]. The increase in SA production occurs in the root following colonization, along with the accumulation of hydrolytic enzymes and ROS, as well as the activation of the phenylpropanoid pathway, albeit at a lower level than the plant–pathogen interaction [129,130]. For a successful establishment of the association, the inhibition of some responses regulated by SA is essential [130]. In this way, PGPM secretory effector proteins interfere with the host plant’s immune system by suppressing SA-dependent defense reactions [131]. The up-regulation of other phytohormones, such as JA, also plays a key role in the creation and maintenance of the mutual association.

Unlike SAR, ISR determines the expression of defense-related genes that are JA- and ET-responsive [117] and does not necessarily imply accumulation of PR (pathogenesis-related) proteins. However, several lines of evidence suggest that the signaling pathways differ depending on the type of PGPM, pathogen, and host plant [132].

Root colonization with *Trichoderma* primes leaf tissues for enhanced activation of JA-regulated defense responses leading to increased resistance to necrotrophic pathogens [117,120,121,133]. Furthermore, changes in both ET/JA and SA pathways were observed in plants inoculated with *T. harzianum* (Table 2), leading to greater resistance to pathogens [134,135].

Sometimes, phyto-friendly soil-microbe-mediated ISR is triggered by increasing sensitivity to hormones rather than by enhancing the expression of JA/ET-responsive genes. Pieterse et al. [106] demonstrated that, after treatment of *A. thaliana* roots with *P. fluorescens* WCS417r, neither the JA content nor the ethylene profile were impaired, but the ability to convert 1-aminocyclopropane-1-carboxylate (ACC) to ethylene was significantly improved, providing greater potential to produce ethylene in the event of a pathogen attack. On the other hand, a greater expression of ET-related genes is likely associated with the host-plant response to root colonization. As previously mentioned, plants can perceive it as a biotic stress [80,136]. For example, *B. cereus* AR156 can induce ISR (Table 2) and, consequently, resistance to *P. syringae* pv. *tomato* DC3000 infection, in *A. thaliana* plants by activation of JA/ET-dependent signaling pathways [107]. Several PGPRs synthesize JA and SA [137]. For example, inoculation of host plants with *P. fluorescens* and *P. aeruginosa* strains (Table 2) resulted in an increase in the endogenous level of SA and, consequently, protection against *Sclerotium rolfsii* infection [108]. 

It has been found that ISR, through the crosstalk with the JA pathway, is activated by VOCs [138]. *A. thaliana* plants exposed to *B. subtilis* strain FB17 showed reduced disease severity against *P. syringae* pv. *tomato* DC3000 because of the activity of the elicitor, acetoin (3-hydroxy-2-butanone), in triggering the ISR [109]. Two VOCs (from *Ampelomyces* sp. and *Cladosporium* sp.) radically reduced disease severity in *A. thaliana* plants challenged by *P. syringae* pv. *tomato* DC3000 (Table 2), going on to affect the expression of genes in the JA pathway [122].

## 5. Case Study: Common Mechanisms in PGPM–Tomato Interactions Highlight Their Behavioral Diversity 

Tomato (*Solanum lycopersicum* L.) is one of the most consumed vegetables and is widely recognized as a model species for basic and applied research on fruit quality and for studies on the genetics and molecular aspects of disease resistance mechanisms [139,140]. PGPMs exert their plant-growth-promoting abilities also on tomato plants through the above-described direct and indirect mechanisms improving nutrient uptake [141,142], (a)biotic stress tolerance [102,143,144,145], and fruit quality and yield [146,147]. 

Given the advantages of applying microbial biostimulants, the molecular basis of the interaction between tomato and phyto-friendly soil microbes has been increasingly studied, mainly because of ‘omics’ approaches. PGPM-mediated global transcriptome, proteome, and metabolome changes in tomato have been recorded primarily at systemic level. Their effect during development and growth, defense against pathogens and abiotic or nutritional stress tolerance have been the focus of several recent articles [118,148,149,150].

**Figure 1:**
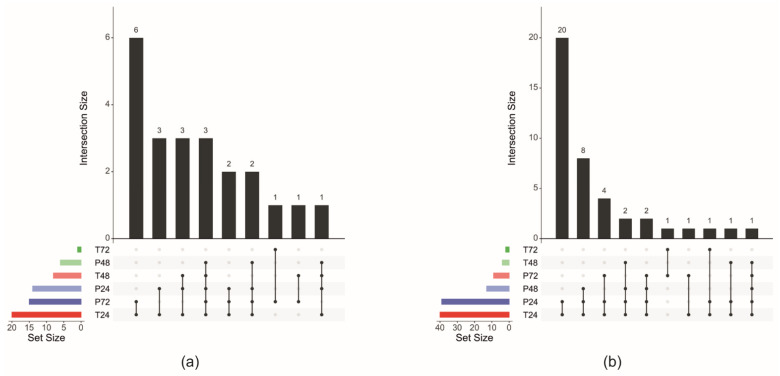
UpSet plot of the intersection of (**a**) up-regulated and (**b**) down-regulated genes during the early interaction of tomato roots with *Trichoderma harzianum* T22 or *Pseudomonas fluorescens* Pf-16. The numbers of genes (black bars) are indicated for each time point (colored bars) intersection. T = *T. harzianum* strain T22; P = *P. fluorescens* strain Pf-16. Tomato roots were sampled at 24, 48, and 72 h post-inoculum.

Despite the efforts of the scientific community, the cascade of molecular events that characterizes the interplay between tomato and phyto-friendly soil microbes at the root level is still poorly documented [20,151]. Recently, two of our articles have provided new information about the molecular mechanisms of the tomato response to the early stages (24, 48, and 72 h post-inoculum, hpi) of root stimulation by *T. harzianum* T22 (T22) or *P. fluorescens* CREA 16 (Pf-16) [17,18]. We started from the list of the identified differentially expressed genes (DEGs). As the fold change (FC) threshold values for the DEG call were different, we first standardized the FC threshold by setting it to ±1.1. After combining the two datasets, we identified genes whose transcriptional activity was regulated in at least one time point in both experiments (Table S1). Then, we focused on the up-regulated (n = 22) and down-regulated (n = 41) genes in at least one time point in both treatments (Figure 1a,b; Tables S2 and S3). Data gathering and integration will allow us to have an overview of the common processes modulated by both microorganisms that are responsible, in part, for their beneficial effects on the plant. Furthermore, a targeted and in-depth study of the most relevant pathways and related genes highlighted differences in terms of host transcriptome reprogramming between the two microorganisms.

### 5.1. Tomato–PGPM Interaction Model for Promoting Nutrient Uptake and Plant Growth 

Plants use several sources of nitrogen in the soil, including inorganic and organic (amino acids) molecules [152]. In our model, T22 and Pf-16 modulate genes involved in the nutrient uptake differently during the first phase of the interaction. Within the first 24 hpi, only two genes encoding for the nitrate transporters were up-regulated in T22-inoculated plants. On the other hand, down-regulation of the genes involved in the nitrate transport was observed at 24 and 72 h in Pf-16-inoculated plants. Conversely, two genes encoding sulphate and ammonium transporters, respectively, were up-regulated in T22- and PF-16-inoculated plants at 24 h (Table S1). 

Increased expression in genes encoding the nutrient transporters is a common trait of plant–PGPM interaction, as shown in Calvo et al. [70] and Singh et al. [71], where a *Bacillus* consortia and *T. asperellum*, respectively, are responsible for the up-regulation of several genes involved in nitrogen uptake. 

Nodulins and nodulin-like proteins play an important role in the plant–PGPM interaction as their expression can be modulated by both bacteria and fungi. Recent studies have highlighted the importance of nodulin-like proteins, in particular in non-nodulating plant species such as tomato, for the nutrient/solutes uptake and in the main aspects of plant growth promotion [153]. As with some nutrient transporters, a down-regulation within the first 24 h was observed in three nodulin-like genes, while an opposite trend was recorded for the nodulin 93 ENOD93 protein (Tables S1 and S3). ENOD93 has been reported to be involved in embryogenesis [154] and in enhancing nitrogen uptake [155].

Hormonal crosstalk was also affected by plant–PGPM interaction, as shown by the down-regulation of genes encoding the auxin response factor at 24 h (Table S3) and the up-regulation along all the time points, particularly in Pf-16-inoculated plants, of auxin efflux carrier genes (Tables S1 and S3). The auxin efflux carrier could be directly involved in IAA transport and in plant-growth promotion during the interaction, as reported in Lewis et al. [156]. On the other hand, due to the crosstalk between the ethylene and auxin pathways in the root [157], a suppression of ET signaling could be suggested by the down-regulation of the auxin response factor genes at 24 hpi.

### 5.2. Tomato–PGPM Interaction Model for the Plant Defense Priming

The early stages of root colonization (within the first 24–48 h) by PGPMs are considered by the plant as an attack. As a result, host plants limit microbial spread in the root through signaling molecules such as ROS and/or calcium, as well as by efficiently activating the SA signaling pathway [127,130]. Accordingly, our combined data showed that SA triggers a reinforcement of the plant cell wall, activating at 24 hpi the expression of key genes involved in lignin (i.e., caffeoyl-CoA O-methyltransferase; Table S1). SA also recruits the defensive arsenal of the tomato plant. We observed the up-regulation of genes encoding antioxidant enzymes (e.g., POXs; Table S1), which when combined with SA-induced defense genes (e.g., wound-induced proteins, chaperon proteins; Table S1) could further help limit microbial spread. It is interesting to note that the induction at 24 hpi of genes encoding strictosidine synthase-like protein and tropinone reductase I (Table S1), belonging to the biosynthetic pathway of alkaloids, highlights a possible role in the tomato defense system of secondary metabolites. Lastly, our data confirmed a remarkable decrease in the ET mediated signaling pathway at 24 hpi, as indicated by the down-regulation of genes encoding ACS and ACO in Pf-16-inoculated plants and of several ethylene-responsive transcription factors in both PGPMs (Tables S1 and S3). 

Conversely, PGPMs have developed ingenious mechanisms to hijack hormone-regulated plant immunity to establish a prolonged mutualistic association [127,130]. To this end, the up-regulation of the gene encoding salicylic acid carboxyl methyltransferase (SAMT) at 24 hpi can help attenuate the SA-based defense pathway through its conversion into the volatile compound, methyl salicylate (MeSA). In addition, at 72 hpi, PGPMs concurred to minimize the level of SA affecting the expression of SA biosynthetic genes (i.e., isochorismate synthase; Table S1). 

Cell wall modifications lead to an intimate relationship between tomato roots and PGPMs. At 24–48 hpi, increased expression of genes encoding lytic or cell-wall loosening agents, such as polygalacturonases (PGs) and expansins (Tables S1 and S2) associated with the down-regulation of several extensins and xyloglucan endotransglucosylases/hydrolases (XHTs), determine the softening and disassembling of the cell wall to aid in the establishment of the interaction. At 72 hpi, the beneficial outcomes of the interaction in terms of growth promotion and priming of plant defense responses begin to be evident. The up-regulation of genes encoding expansins or XHTs in Pf-16 and T22-inoculated plants promotes cell enlargement, thus favoring root developmental processes. Instead, induction of SA-related defense genes (e.g., dehydroascorbate reductase 1, DHAR1; HSP; universal stress protein, USP; Tables S1 and S2) allows the plant’s defense system to be kept in a pre-alert state, which makes the plant able to respond more quickly and effectively against a subsequent biotic attack. 

## 6. Conclusions 

**Figure 2:**
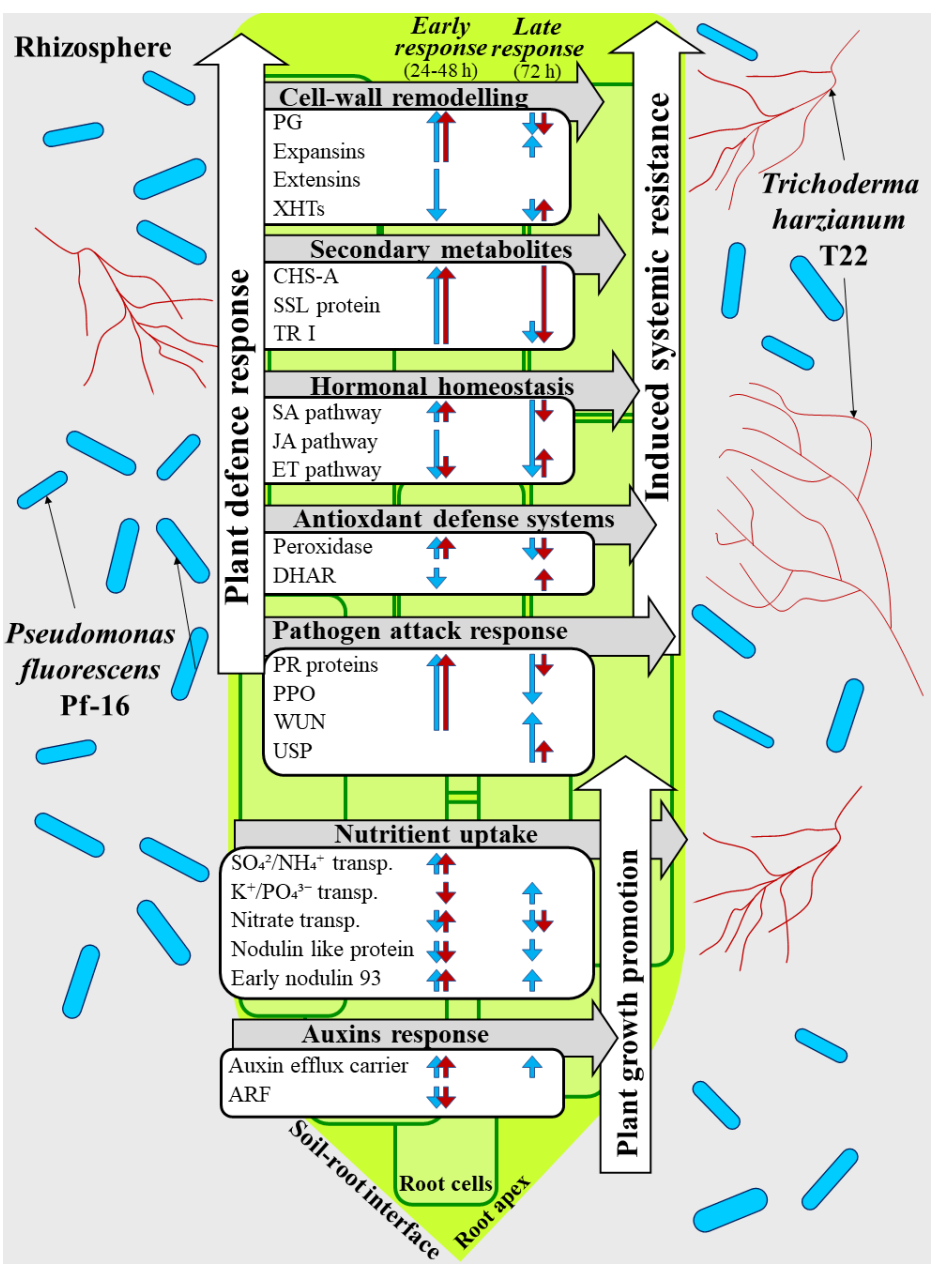
Model of the early molecular events occurring in the tomato root–PGPM interaction. Overview of tomato root transcriptomic responses after inoculum with *Trichoderma harzianum* T22 and *Psuedomonas fluorescens* Pf-16. The highlighted functions derive from the annotation of differentially expressed genes as reported in De Palma et al. [17] and Scotti et al. [18]. Tomato genes that were significantly up-regulated and down-regulated in response to *T. harzianum* T22 or *P. fluorescens* Pf-16 at each time point (24, 48, and 72 h post-inoculum) are in red and blue, respectively. PG: polygalacturonases; CHS-A: Chalcone synthase-A; SSL: Strictosidine synthase-like; TR: tropinone reductase; SA: salicylic acid; JA: jasmonic acid; ET: ethylene; DHAR: dehydroascorbate reductase 1; PR: pathogenesis-related; PPO: polyphenol oxidase; USP: universal stress protein; ARF: auxin responsive factor.

The use of phyto-friendly soil microbes in agricultural practice and research has greatly increased because of their multiple traits [158]. Microbial-based biostimulants represent a relevant challenge in sustainable agriculture that requires increases in yield and product quality, while reducing the negative impact of agrochemicals on the environment [5]. Furthermore, more sustainable agriculture could be fostered by microbial-based biostimulants to strengthen the innate immune system of plants, thus preventing the extent and frequency of emerging plant diseases. However, the practical use of PGPMs is often hampered by relatively poor plant growth and disease control and all known constraints associated with their large-scale use. This variability in their effects is strongly influenced by the genotype of the host plant, by sophisticated nutritional and chemical signals, as well as by pedoclimatic factors. Understanding the induction of plant responses by phyto-friendly soil microbes is essential for the development of novel strategies for managing plant growth and diseases. High-throughput technologies and ‘omics’ sciences have given a great impetus toward understanding processes modulated by PGPMs, which ultimately contribute to beneficial effects on plant development, fitness, health, and adaptation to environmental perturbation. Based on the recent literature and on the main findings of our previous studies [17,18], a model describing the host plant’s molecular responses across the experimental period analyzed (24-48-72 hpi) is here proposed (Figure 2). Data gathering highlighted the processes that are crucial for the beneficial outcome of the interaction. In the early phases of plant response (24–48 hpi), SA signaling combined with inhibition of JA- and ET-biosynthesis activate the plant’s defense machinery to limit PGPM spread. At 72 hpi, an up-regulation of hormone-related genes has allowed the tomato plant’s defense system to be kept in a pre-alert state, which makes the plant able to respond more quickly and effectively against a subsequent biotic attack. 

Pf-16- and T22-induced plant growth stimulation begin to be evident already at 24 hpi. PGPMs improve the nutrient uptake capacity of tomato mainly through a greater assimilation of sulphate and the involvement of nutrient transporters. As for NUE, data highlighted a behavioral difference between Pf-16 and T22. *Trichoderma* T22 seems to confirm its ability to enhance the capacity of nitrogen use in plants [48], also through a specific regulation of NRT/AMT transporters. In addition, the up-regulation of genes responsible for cell-wall loosening and modulation of ET/auxin signaling at 72 hpi could affect root architecture by promoting root developmental processes. 

All the information gathered from the literature overview and from the interaction model represented in Figure 2 will support the research community to expand their knowledge of host-plant responses to the interaction with phyto-friendly bacteria and fungi and will help define new strategies for the selection of crop varieties with a better ability to take advantage of microbial stimulation.

## Figures and Tables

**Table 1:** Different mechanisms of plant growth promotion used by PGPMs.

	Mechanisms	Beneficial Effects	References
**PGPR**			
*Azotobacter* spp., *Azospirillum amazonense*	Biological N_2_ fixation (BNF)	Increased grain dry matter accumulation, yield	[39,40]
*Pseudomonas extremaustralis*, *Bacillus licheniformis*, *Enterobacter asburiae*	Phosphates (P)-solubilization	Increased root and shoot biomass and P concentration	[41,42]
*Pseudomonas fragi,* *Pantoea dispersa, P. agglomerans, Enterobacter cloacae,* *Rhizobium* sp.	Zinc-solubilization	Increased shoot and root dry weights as well as shoot and root lengths	[43]
*Pseudomonas fluorescens*	Siderophores	Improved plant growth, increased iron inside plant tissues	[44]
*Bacillus* sp. LZR216	Enhance auxin biosynthesis	Root development	[45]
*Burkholderia phytofirmans*	Induction of auxin efflux root transporters genes	Primary root growth	[46]
*Aneurinibacillus aneurinilyticus*, *Paenibacillus* sp.	ACC deaminase activity	Increased tolerance to salinity stress	[47]
*Pseudomonas fluorescens* SS101, *Bacillus subtilis* SYST2	Production of volatile organic compounds	Increased plant biomass	[34,37]
**PGPF**			
*Trichoderma harzianum* T22, *Trichoderma virens* GV41	Nitrogen use efficiency (NUE)	Increased yield and biomass production	[48,49]
*Aspergillus* sp. NPF7	Phosphates (P)-solubilization	Increased root and shoot length	[50]
*Trichoderma harzianum* T-203	Minerals uptake	Biomass accumulation, increased concentration of microelements	[51]
*Aspergillus fumigatus* TS1, *Fusarium proliferatum* BRL1	Production of indol-3yl-acetic acid and gibberellic acid	Higher chlorophyll content, root-shoot length, and biomass production	[52]
*Piriformospora indica*	Increased auxin levels	Root elongation	[53]
*Trichoderma harzianum, Trichoderma atroviride*	Auxin-like compound production	Plant growth regulation	[54]
*Trichoderma asperellum T203*	ACC deaminase activity	Root elongation	[55]
*Trichoderma asperelloides*	ACC deaminase activity	Increased tolerance to salinity stress	[16]
*Trichoderma virens* Gv. 29-8	IAA and auxin exogenous production	Biomass, lateral root development	[56]
*Trichoderma* spp.	Production of volatile organic compounds	Increased plant growth and modification of root architecture	[38]
*Phoma* sp. GS8-3	Production of volatile organic compounds	Increased plant growth	[57]

**Table 2:** Role of PGPMs in induced systemic resistance.

	Mechanisms	Beneficial Effects	References
**PGPR**			
*Bacillus pumilus* INR-7	Enhancing of cell wall lignification	Disease protection against *Sclerospora graminicola* infection	[100]
*Pseudomonas aureofaciens* 63–28	Induction of PAL, POX and PPO enzymes	Disease protection against *Pythium aphanidermatum* infection	[101]
*Bacillus subtilis* MBI600	Induction of pathogenesis-related genes	Disease protection against *Rhizoctonia solani*, *Pythium ultimum*, and *Fusarium oxysporum* f.sp. *radicis-lycopersici* infection	[102]
*Paenibacillus alvei* K165	Induction of pathogenesis-related genes	Disease protection against *Verticillium dahlia* infection	[103]
*Pseudomonas fluorescens* CHA0 *Pseudomonas aeruginosa* 7NSK2	Induction of phytoalexins production	Disease protection against *Botrytis cinerea* infection	[104]
*Pseudomonas fluorescens* SS101	Induction of genes involved in camalexin and glucosinolates biosynthetic pathways	Disease protection against *Pseudomonas syringae* pv *tomato* and *Spodoptera exigua*	[105]
*Pseudomonas fluorescens* WCS417r	Enhanced capacity to convert ACC to ethylene	Enhanced resistance to *Pseudomonas syringae* pv. *tomato* DC3000	[106]
*Bacillus cereus* AR156	Activation of the salicylate- and jasmonate/ethylene-signaling pathways	Induced resistance against *Pseudomonas syringae* pv. *tomato* DC3000	[107]
*Pseudomonas fluorescens* Pf4 *P. aeruginosa* Pag	Phenolic compounds production stimulation and ISR via salicylate-dependent pathway induction	Induced resistance against *Sclerotium rolfsii* infection	[108]
*Bacillus subtilis* FB17	Induction of ISR via salicylate/ethylene pathway	Reduction disease severity against *Pseudomonas syringae* pv. *tomato* DC3000	[109]
**PGPF**			
*Trichoderma* NBP-67 *Aspergillus* NBP-08, *Penicillius* NBP-45, *Talaromyces* NBP-61, *Trichoderma* T-42, MV-41, DFL, RO, *Trichoderma atroviride* TRS25	Enhancing of cell wall lignification, callose deposition	Disease protection against and *Colletotrichum capsici*, *Fusarium oxysporum* f. sp. *Ciceris*, *Rhizoctonia solani* infection	[110,111,112]
*Trichoderma hamatum* Th23	Induction of genes for biosynthesis of phenolic compounds and activation of pathogenesis-related genes	Disease protection against *Tobacco Mosaic Virus* infection	[113]
*Paenibacillus dendritiformis* *Trichoderma harzianum* *Trichoderma asperellum*	Enhancing the activity of defense-related and antioxidant enzymes	Disease protection against *Colletotrichum truncatum* infection	[114]
*Aspergillus fumigatus* *Rhizopus oryzae*	Enhancing the activity of defense-related enzymes	Disease protection against *Fusarium Wilt* infection	[115]
*Trichoderma harzianum* TH58	Induction of Ascorbate-Glutathione cycle (Asa-GSH) and oxidative pentose phosphate pathway (OPPP)	Disease protection against *Fusarium oxysporum* infection	[116]
*Trichoderma harzianum*	Induction of genes for biosynthesis of phenolic compounds	Disease protection against *Botrytis cinerea* infection	[117]
*Trichoderma harzianum* T22	Induction of defense-related genes; modulation of phenylpropanoids pathway	Disease protection against *Macrosiphum euphorbiae* infection	[118]
*Penicillium chrysogenum* PenC-JSB9	Induction of defense-related genes	Disease protection against *Sclerospora graminicola* infection	[119]
*Trichoderma atroviride* P1 *Trichoderma harzianum* T22	Up-regulation of the salicylic acid pathway	Disease protection against *Botrytis cinerea* infection	[120]
*Trichoderma harzianum*	Enhanced JA-dependent defense responses	Resistance induced against *Botrytis cinerea*	[121]
*Ampelomyces* sp. *Cladosporium* sp.	Induction of ISR via jasmonate/ethylene pathway	Reduction disease severity against *Pseudomonas syringae* pv. *tomato* DC3000	[122]

## Data Availability

Not applicable.

## Supplementary Materials

The following supporting information can be downloaded at https://www.mdpi.com/article/10.3390/plants11202672/s1, Table S1: Complete list of common DEGs retrieved from De Palma et al. [17] and Scotti et al. [18]. For each gene at different time points, namely 24, 48, and 72 h post-inoculum (hpi), it is reported the expression level estimate (Log_2_ Fold-change) and a statistical significance value as calculate by edgeR. Merged datasets were filtered identifying those genes whose transcriptional activity was regulated in at least one time point in both experiments, using a standardized fold change of ±1.1 and FDR < 0.05; Table S2: List of common up-regulated DEGs retrieved from De Palma et al. [17] and Scotti et al. [18]. For each gene at different time points, namely 24, 48, and 72 h post-inoculum (hpi), it is reported the expression level estimate (Log_2_ Fold-change) and a statistical significance value as calculate by edgeR. Merged datasets were filtered identifying those genes whose transcriptional activity was regulated in at least one time point in both experiments, using a standardized fold change of ±1.1 and FDR < 0.05; Table S3: List of common down-regulated DEGs retrieved from De Palma et al. [17] and Scotti et al. [18]. For each gene at different time points, namely 24, 48, and 72 h post-inoculum (hpi), it is reported the expression level estimate (Log_2_ Fold-change) and a statistical significance value as calculate by edgeR. Merged datasets were filtered identifying those genes whose transcriptional activity was regulated in at least one time point in both experiments, using a standardized fold change of ±1.1 and FDR < 0.05.
